# Blurring the boundary between models and reality: Visual perception of scale assessed by performance

**DOI:** 10.1371/journal.pone.0285423

**Published:** 2023-05-08

**Authors:** Tim S. Meese, Daniel H. Baker, Robert J. Summers

**Affiliations:** 1 College of Health and Life Sciences, Aston University, Birmingham, United Kingdom; 2 Department of Psychology and York Biomedical Research Institute, University of York, York, United Kingdom; Justus Liebig Universitat Giessen, GERMANY

## Abstract

One of the primary jobs of visual perception is to build a three-dimensional representation of the world around us from our flat retinal images. These are a rich source of depth cues but no single one of them can tell us about scale (i.e., absolute depth and size). For example, the pictorial depth cues in a (perfect) scale model are identical to those in the real scene that is being modelled. Here we investigate image blur gradients, which derive naturally from the limited depth of field available for any optical device and can be used to help estimate visual scale. By manipulating image blur artificially to produce what is sometimes called fake tilt shift miniaturization, we provide the first performance-based evidence that human vision uses this cue when making forced-choice judgements about scale (identifying which of an image pair was a photograph of a full-scale railway scene, and which was a 1:76 scale model). The orientation of the blur gradient (relative to the ground plane) proves to be crucial, though its rate of change is less important for our task, suggesting a fairly coarse visual analysis of this image parameter.

## Introduction

Our visual perceptions begin with a flat two-dimensional (2D) image from which we must extract the embedded third dimension (see [1] for a recent review and [2] for a recent proposal). We are readily able to judge the scale (the physical size of the grain) of novel environments and the depth relations within them, even if they don’t contain familiar objects (e.g. [3–5]), so how might the necessary estimates of relative and absolute depth be achieved?

A wealth of visual cues is available for this task, and it has long been recognized that gradients of various image measures provide a valuable source of quantitative information about the three-dimensional (3D) world. One important class of depth cues (we shall call them ‘class 1’) includes those pictorial cues—cues that can be used by artists to convey depth on their two-dimensional (2D) canvas—for which the relevant image measures diminish towards a vanishing point on the horizon, regardless of what part of the scene is being fixated (e.g., linear perspective, size- and texture-gradients, height in the visual field). Under appropriate viewing conditions, well-constructed pictorial images can produce compelling impressions of depth (e.g., see the artwork of Vasarely), comparable to that achieved through binocular stereopsis and motion parallax (see also [6]). These perspective cues can provide valuable information about relative depth (e.g., one object is twice as far away as another) but provide no information about absolute depth (distance) or scale. For example, the texture and linear perspective cues in a real scene are identical to those in a small-scale model of that scene. For the problem of scale to be solved, there must be an estimate of absolute depth for at least one point in the scene.

A second class of depth cues (we shall call them ‘class 2’) is those for which the relevant image measures diminish towards the point of static fixation (horizontal binocular disparity, motion parallax) and/or focus (defocus blur). Taken alone, these cues are even more impoverished than class 1 cues and provide only ordinal information (e.g., one object is further away than another—you also know the relative distances of the two objects to the horopter, but since you don’t know where that is, that is not very helpful.); but in conjunction with information about the distance to the point of fixation, from which the depth of everything else can be judged, they become much more valuable and can be used to recover absolute depth across (large parts) of the scene. However, the problem here is that the entire strategy is critically dependent on the estimated distance to the point of fixation. This task is traditionally attributed to the ocular-motor cues of accommodation and vergence angle, but these are very poor measures beyond 2 or 3m or so, and so the whole enterprise becomes unreliable. Another possibility is to use vertical disparity [7], but for this to work a large field of view of a surface is needed [8]. Familiarity (e.g., knowledge about the size of people, for example) might be used to solve the problem (e.g. [9]), but in fractal scenes (e.g., a seashore scene), familiar objects are not always to be found. Fortunately, there are other strategies that can also be adopted [10–13]. Mathematical work by Okatani and Deguchi [14] and Held et al. [13] has shown that an intersection of constraints between the class 1 and class 2 cues described above can be used to compute absolute depth information. This mathematical work was directed at the class 2 cue of defocus blur, but since the core mathematics is identical to that for horizontal binocular disparity [15] and motion parallax (setting aside that defocus blur is unsigned), its implications can be extended to all three class 2 cues. A natural question then has been whether human vision is able to exploit this type of image analysis to estimate scale.

Demonstrations of so-called *tilt shift miniaturization* (e.g., [16]), where the addition of artificial blur gradients to (carefully selected) natural scenes is found to shrink their perceived scale, suggest that the visual system can do something along these lines. These media and other demonstrations [13, 17] were the initial motivating influence of our experimental enquiry here and we will return to them in the Discussion. In what follows then, we concentrate on the role of the class 2 blur cue. The reason that defocus blur serves as a depth cue is that imaging devices (including the eye) have a limited depth of field: objects in the plane of focus are imaged sharply, but objects and surfaces that are closer or further away are blurred. The severity of defocus blur increases linearly with distance from the plane of focus, producing gradients of blur across the image [18]; and because image blur depends on depth it can, in principle, be used to recover it [19]. In fact, experiments have shown that human vision can exploit this, at least when judging ordinal depth [20–24] or surface slant [25]. Furthermore, depth of field decreases as the focal plane is brought forward in the scene, which means that as that happens, defocus blur increases more rapidly with distance from the focal plane. This suggests the possibility of a more heuristic approach to the problem than the intersection of constraints solution proposed by Held et al. [13]: steep blur gradients in the image might be taken as a cue to nearness, from which scale might then be judged [12, 17]. However, our aim was not to distinguish empirically between these different approaches to determining scale [13, 17], the point being that each has *prima facia* plausibility for the visual system. Instead, our focus was on devising a clean experiment that was able to assess the role of blur gradients in the tilt shift miniaturisation phenomenon.

We know of only two studies that have attempted to investigate this potentially important blur gradient cue in this context, and both involve methodological problems. (We consider a third study, [26], in the Discussion, since its relation to ours is more by stimulus choice than motivation.) Held et al. [13] presented their participants with GoogleEarth images that were subject to various blur treatments and asked them to judge the perceived distance from the camera to a target building identified in the image. However, this subjective numerical estimation task was clearly prone to error, since the authors rejected 30% of their participants for using the ‘wrong’ strategy. A study by Vishwanath and Blaser [17] improved on the methodology by using a series of gauge figures (a gloved hand, held against a stone wall, photographed from various distances) to match the perceived distance to images of rock faces subjected to various blur gradients and other treatments, such as angle of surface slant and tilt. However, this task still involved the subjective evaluation of distance, and it is unclear, for example, what type of strategy observers would have adopted when the perceived tilts and/or slants of the full field surfaces being compared were different (e.g., against which part of the surfaces were the distance judgements being made?). Both studies reported that blur gradients affected the perception of absolute depth, but in contrast to Held et al. [13], Vishwanath and Blaser [17] found that the orientation of the blur gradient (whether it was aligned or orthogonal to the underlying surface slant) was unimportant. Unfortunately, however, since there was no condition of uniform blur, it is not clear whether the inclusion of blur *gradients* was the important factor in their study—it is possible, in principle, that merely blurring their images was sufficient to achieve their subjective effects. This would also explain the failure to find a blur orientation effect.

To overcome the problems above we devised a novel way of addressing the question at hand. First, we reasoned that the central issue is the perception of scale and so (unlike the other two studies) we set out to assess this directly. One advantage of this approach is that estimates of scale involve judgements about the entire image and so one might expect it to be more robust than depth estimates about small parts of an image, which may or may not be blurred, for example [13]. Scale is also a single measure of the entire scene, and so one might expect it to be more robust than a distance judgement of a scene, where perceived point distance varies across the scene being judged, as for Vishwanath and Blaser [17]. Second, we recognized that objective psychophysical data on the matter would be available only by using performance measures, where ground truth would allow a formal measure of sensitivity (d-prime).

To meet our design criteria, we devised a two-alternative, forced-choice (2AFC) task in which our participants made judgements about which one of two images was a photograph of a full-scale scene (subject to various blur treatments, including full field blur) and which was a small (1:76) scale model. (This method should not be confused with single interval binary-choice (SIBC). In 2AFC, as defined by Green and Swets [27], there is ground truth, and the results provide an objective performance measure that relates to the observer’s sensitivity to the target which is randomly allocated to one of the two response intervals. In SIBC, the task is often one of classification where there is a single stimulus presentation and two response categories. This method has its place (e.g. when measuring a point of subjective equality) but is inherently subjective and is prone to response bias when applied in detection tasks.) We chose to use British railway scenes as the subject matter of the photographs since the relevant materials were conveniently available. Note, however, that we were not assessing categorical perception, but visual performance: our analysis was based on whether observers were correct, not their subjective categorization.

In sum, our aims were as follows: we wanted to use a forced-choice psychophysical method to give us performance measures of scaling effects in blurred images, specifically those associated with tilt shift miniaturization. Although informal demonstrations of these effects are widespread, if successful, our approach would be the first objective experimental demonstration of them (previous subjective assessments being prone to potential biases). Further, we wished to use our approach to assess the effectiveness of various blur image treatments that might be applied.

## Methods

### Stimuli

The untreated stimuli were grey level images (8 bit deep) that were cropped to 418 pixels wide and 298 pixels tall. They consisted of photographs of British railway scenes containing diesel locomotives. Six of these were photographs of real full-scale scenes, and six were photographs of small-scale (1:76) model scenes. The images for the full-scale scenes were obtained from the Internet; attributions are given in the caption to Fig 4. Our selection criteria required that these images contained (i) a distinct ground plane with optical slant that decreased with distance from the photographer and was (fairly) flat from left to right, (ii) few details that would have been difficult to produce in model form (thereby identifying the image as full scale, for example, smoke or steam, and close-ups of people) and (iii) that the image quality (e.g. focus) was good. The photographs of the exhibition standard model-scenes were taken using a digital SLR with strong indoor lighting and a long exposure (several seconds), permitting the smallest aperture possible with the equipment available. This helped to reduce defocus blur in the model images, which would have been a strong cue to their true scale. The model images were carefully cropped to ensure that they contained no signs of the full-size setting in which they were photographed. The six model images were selected from a larger set of 14 images, being those that looked most realistic according to informal assessment by several volunteers.

We processed the images in Matlab to convert them to achromatic 8-bit images and to adjust their grey levels so that each image had a mean grey level of 127. We then imposed various blur treatments. None was exactly equivalent to the defocus blur that would arise naturally, but the computational analysis of Held et al. [13] has shown that linear blur gradients applied to photographs like those used here (with a distinct ground plane) produce distributions of blur that are consistent with the simulated focal distance.

**Fig 1:**
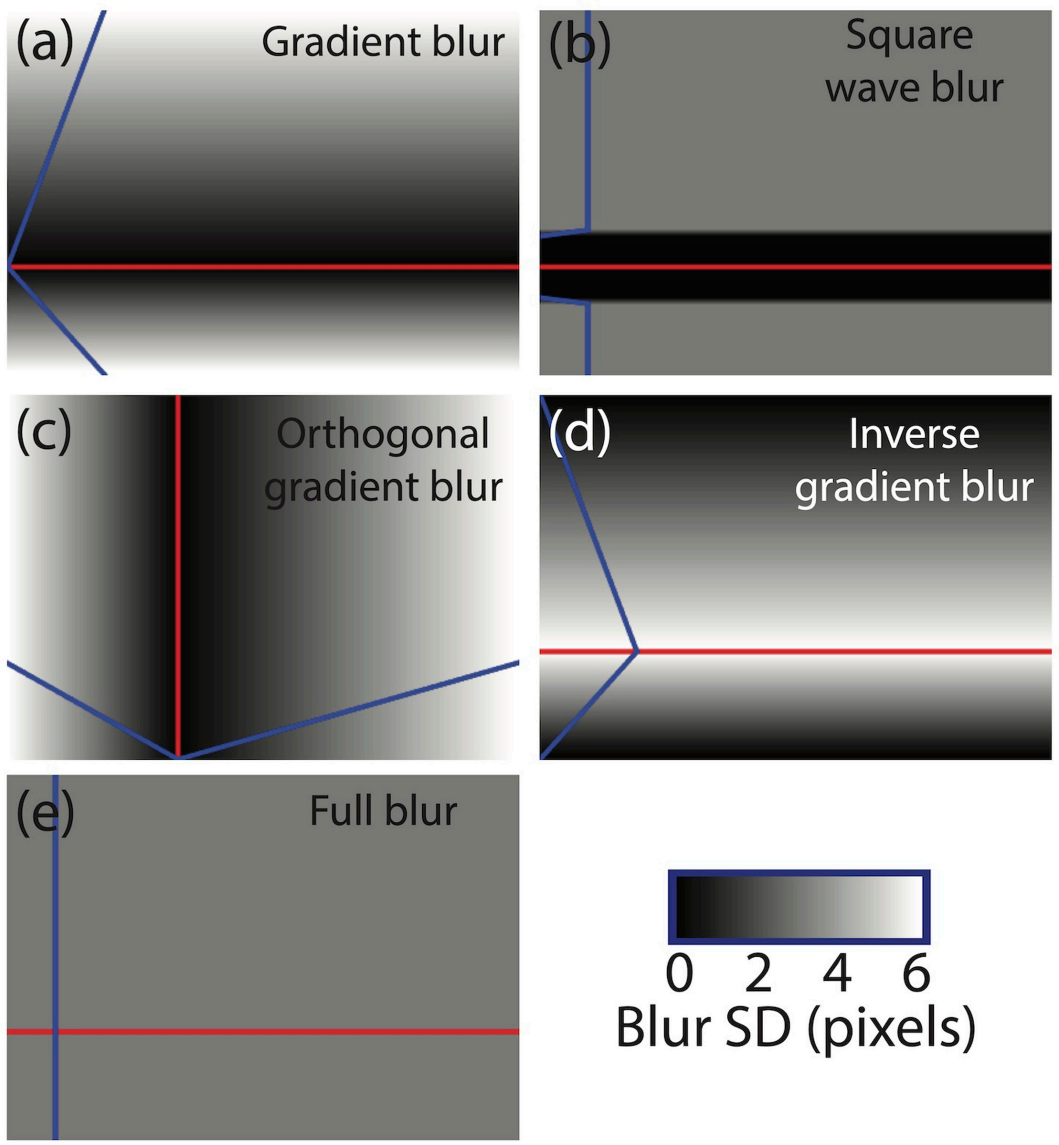
Schematic illustrations of the five blur treatments applied to each of the six photographs of full-scale railway scenes. A sixth condition involved no blur treatment. The red line indicates the contour of focus (the precise position of this varied across the six different scenes). The grey levels (given by the bar in the lower right corner) and blue lines indicate the variation of Gaussian blur across the image. In (a, b, d) the blur was constant horizontally and varied vertically. In (c) the blur was constant vertically and varied horizontally. In (e) the blur was uniform across the entire image. See text for further details. The treatment in (a) is sometimes referred to as fake tilt-shift miniaturization.

**Fig 2:**
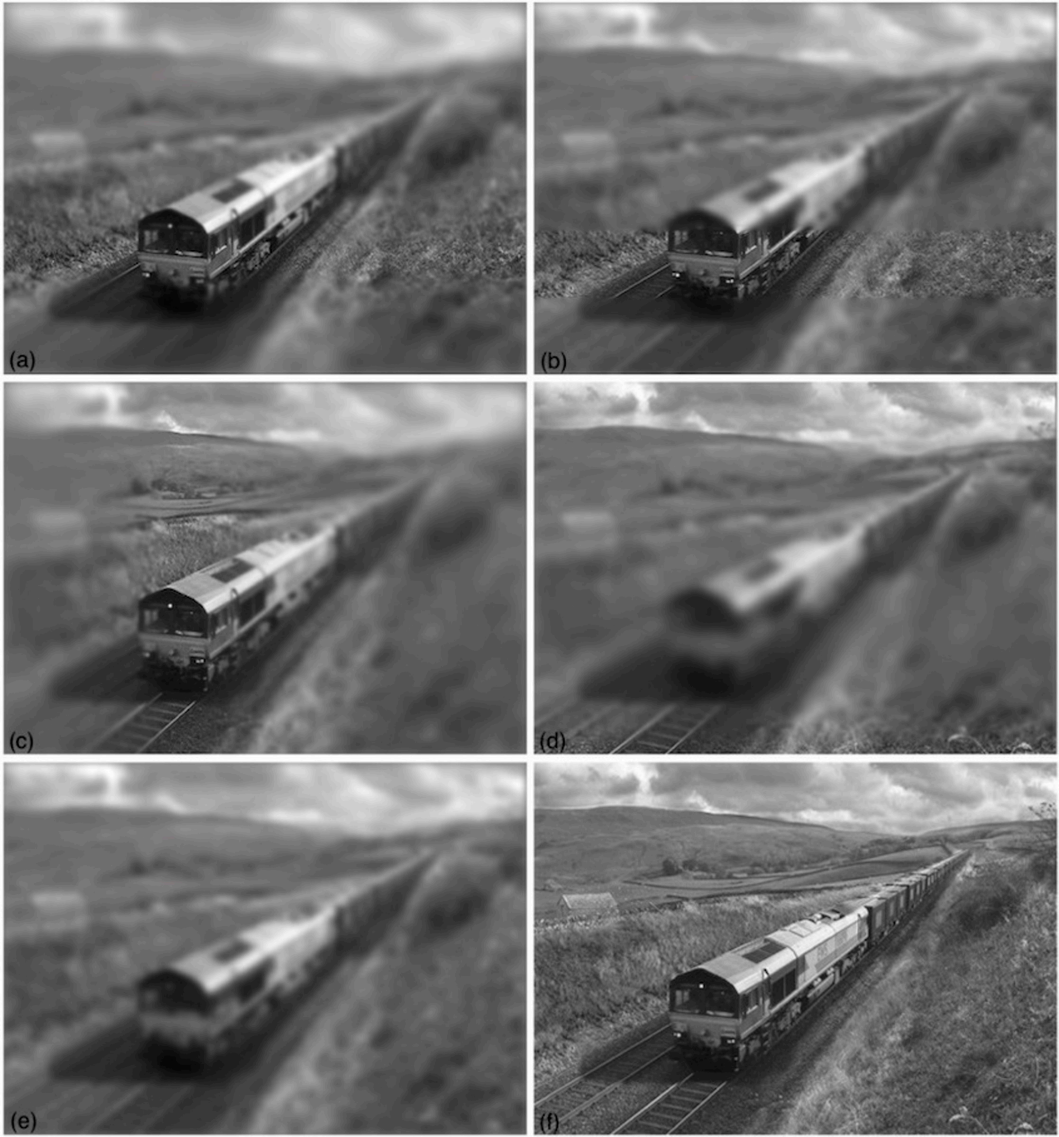
(a-e) The five image treatments described in Fig 1 applied to one of the images of a full-scale railway scene. (f) The original image (photograph by Don Burgess). See the Acknowledgements section for a link to the full set of stimuli used in the experiment.

**Fig 3:**
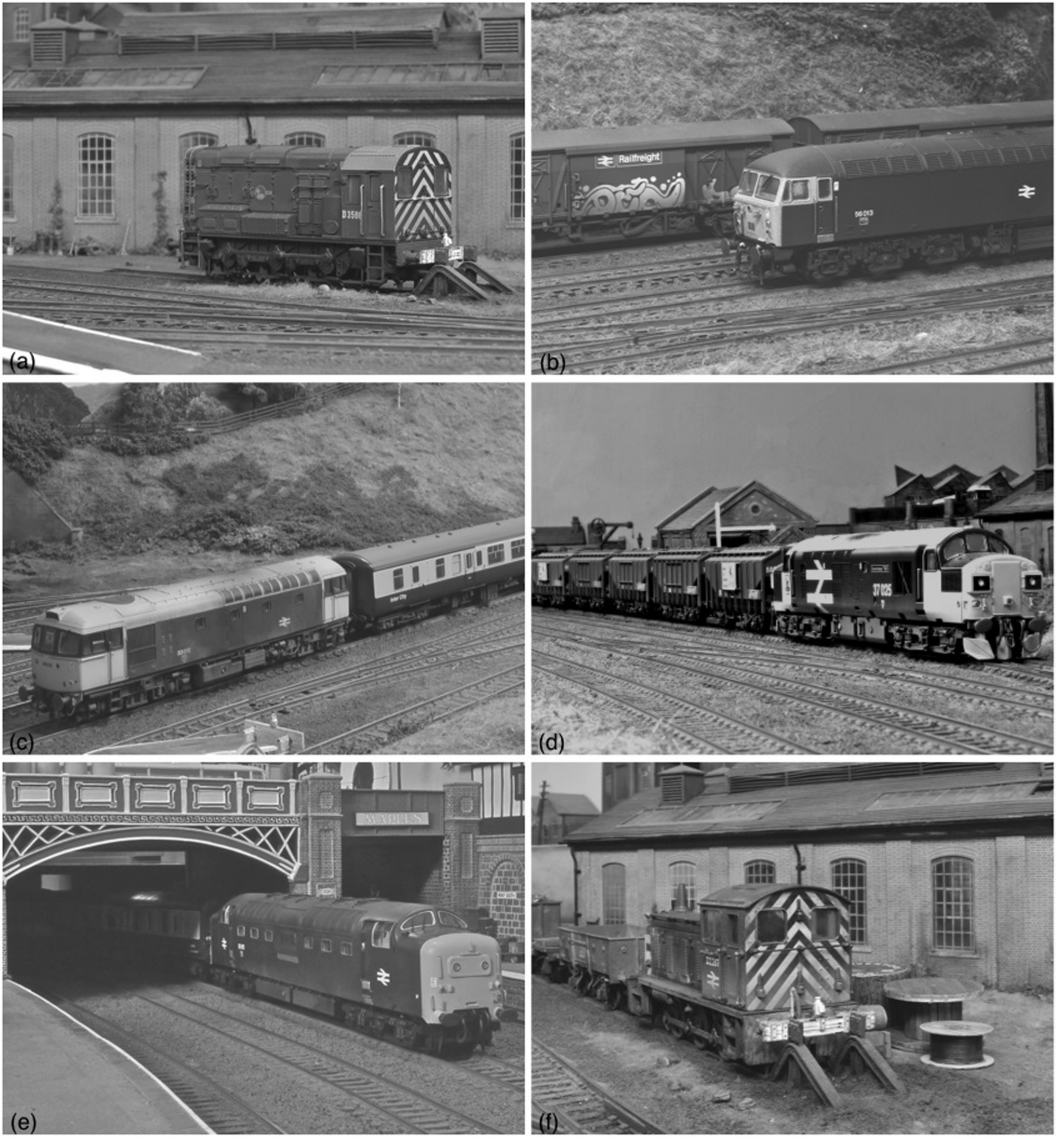
The six scale model images.

**Fig 4:**
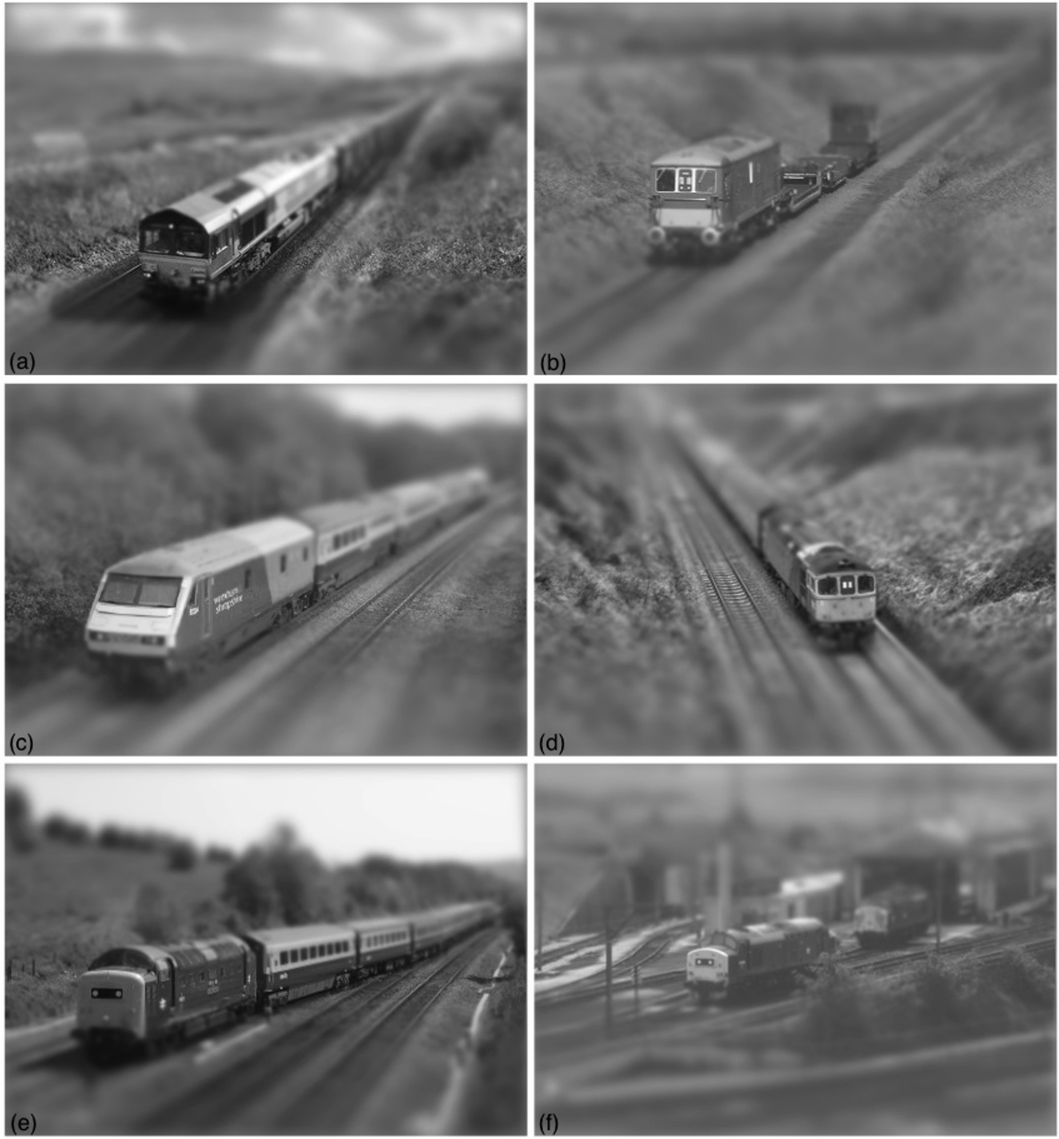
The application of blur treatment from Fig 1A (sometimes known as fake tilt-shift miniaturization) to each of the six full-scale railway scenes. The original images were published under a creative commons license by the following authors: (a) Don Burgess, (b) Alan Murray-Rust, (c) Andy F, (d) Ingy the Wingy, (e) Phil Sangwell, (f) Roger Geach.

Each of the six full-scale images was subject to each of six blur treatments. In a ‘no blur’ treatment, no further image processing was performed. In the other treatments, a *contour of focus* was selected by hand to pass through the front part of the locomotive cab and a circular Gaussian blur kernel was constructed whose maximum blur had a standard deviation of 6 pixels. (A Sinc function is probably a better approximation to the modulation transfer function of the eye’s optics [28] and has been used successfully in experiments employing artificial blur gradients [29]. However, a Gaussian function is commonly used in visual psychophysics (e.g. see [30] for a review) and its use here is unlikely to be critical for our results.) In a ‘gradient blur’ treatment, the blur decreased linearly from its maximum at the bottom of the photograph to zero at the contour of focus, and then linearly back up to its maximum at the top of the photograph (Fig 1A). In a ‘square-wave blur’ condition a horizontal strip of the image (50 pixels wide) was centred on the contour of focus and remained untreated. Above and below this were narrow ramps (5 pixels wide) in which the blur gradient increased from zero to half of its maximum. The remainder of the image was treated with half-maximum blur (see Fig 1B). The purpose of the narrow ramps was to remove the sharp boundary from blur to no blur, which tended to make the images look as though they were being viewed through occluding ‘frosted glass’ with a gap across the middle. An ‘orthogonal gradient blur’ treatment was the same as the gradient blur treatment, except that it was applied horizontally instead of vertically (Fig 1C). In an ‘inverse gradient blur’ treatment the blur gradient was applied in the opposite direction from the gradient blur treatment (Fig 1D) and in a ‘uniform blur treatment’, the entire image was subjected to a fixed level of blur that was half of its maximum (i.e., a standard deviation of 3 pixels). Note that the space-averaged level of applied blur was identical in all the blur treatments except the square-wave condition, which was a little less owing to the unblurred horizontal strip. The result of each of the six treatments is shown for a single full-scale scene in Fig 2. The six model railway scenes are shown in Fig 3, and the application of blur treatment (a) (from Fig 1) is shown for each of the full-scale scenes in Fig 4. A link to the full set of stimuli used in the experiment can be found in the Acknowledgements section.

The value of maximum blur that we chose (standard deviation = 6 pixels) was not derived from mathematical considerations of the scale that we intended to simulate [13]. To do so would require knowledge of the true depth maps of the full-scale photographic images and these were not available to us. Instead, we settled for using a level of maximum blur that produced satisfactory effects according to informal assessment.

### Experimental design

Application of the blur treatments above produced 36 different full-scale images. Each of these was paired with each of the six small-scale model images producing 216 different image pairs. Each participant saw 6 pairs of these images such that they saw all 6 model photographs, all 6 full-scale photographs and all 6 treatments. With this arrangement 36 participants were needed for complete viewing of the full set of image pairs. Note that on each experimental trial, one of the images was a real scene subject to one of the blur treatments (Fig 1), including no blur, and the other was a scale model scene which was not blurred. The task was to select the full-scale image and the correctness of response was recorded.

### Participants

We recruited 108 participants (41 male, 67 female) such that three different participants saw each image pair (see above). Participants were undergraduate optometry students studying at Aston University (average age = 20.7 years). Signed consent was obtained from each participant and the experiments were performed in accordance with the sixth revision of the Declaration of Helsinki (2008) and the ethics committee of the School of Life and Health Sciences at Aston University. It took each participant approximately one minute to complete the experiment. The data were gathered in 2011.

### Procedure and equipment

Stimulus pairs were displayed on a laptop computer (with a maximum luminance of 300 cd/m^2^) one above the other at a comfortable working distance that was fixed throughout the experiment (typically, 60 to 70 cm). This produced image pairs that subtended approximately 12 x 17.7 deg. The order of the six trials was determined randomly for each participant. Whether the image of the full-scale scene was displayed at the top or bottom of the display was determined randomly each trial. At the beginning of the session, each participant was told they would see a short sequence of image pairs depicting railway scenes. One would always be a full-scale scene, the other would always be a small 1:76 scale model. Their task was to decide which one of the images (top or bottom) was the real, full-scale scene. Participants viewed the stimuli binocularly and made their responses by pressing one of two keys on the keyboard of the laptop. Stimulus duration was five seconds, during which time participants were free to move their eyes. Response time was unlimited but in practice a few seconds. The experiment was conducted in informal settings but free from interruptions.

## Results

**Fig 5:**
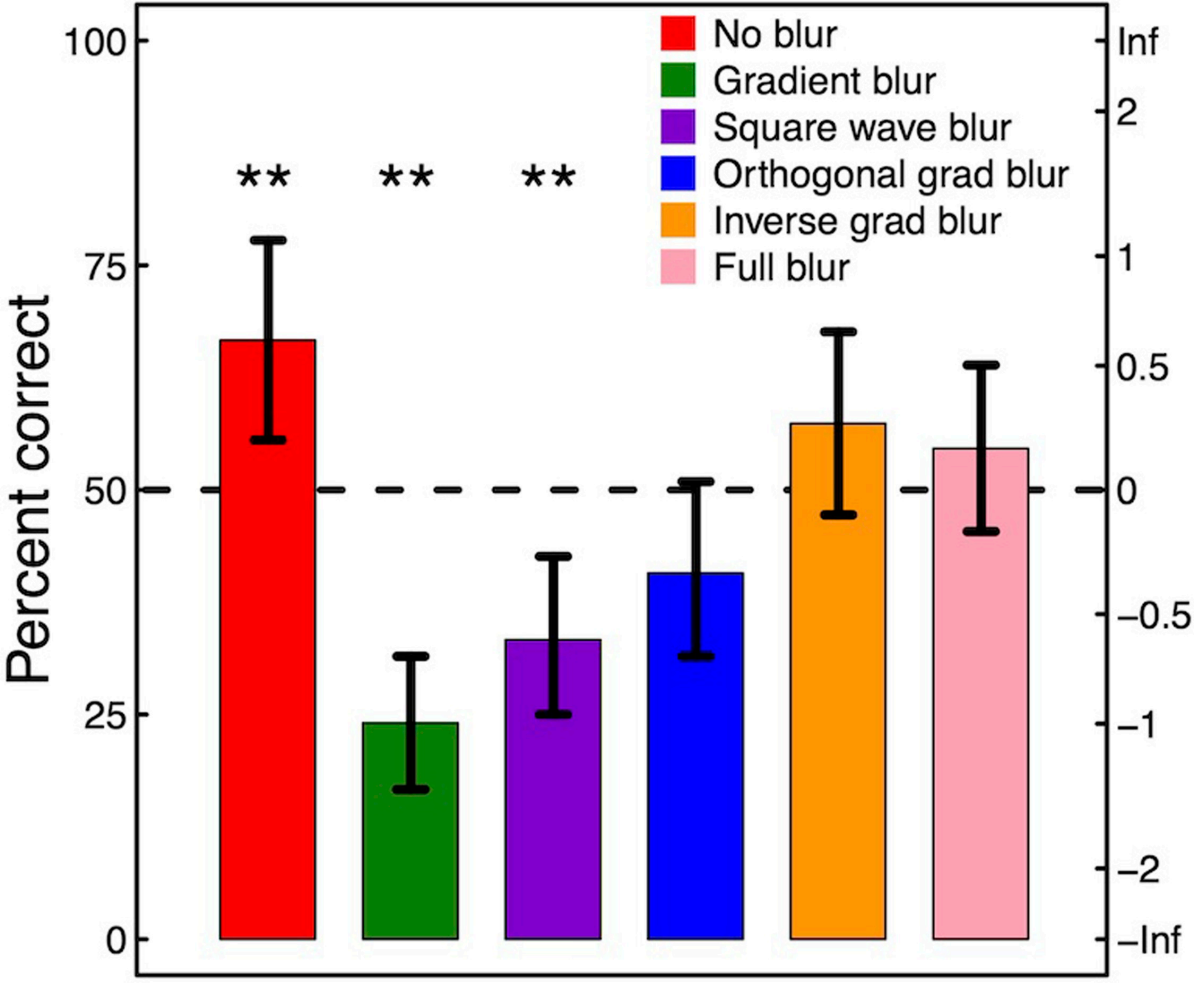
Experimental results: The percentage of trials in which the full-scale scene was correctly identified. Error bars show 95% confidence limits on the mean triplet scores (*n* = 36) determined by bootstrapping. The double asterisks (**) indicate the conditions that were significantly different from chance (see text for statistical details). Responses in the gradient blur condition (green bar) were significantly less often correct than each of the other conditions except for the square wave blur condition (purple bar). See text for details.

The results are plotted in Fig 5 and show the percentage of trials that were correct (left ordinate), pooled across the 108 participants for each of the six blur treatments. Note that for 2AFC, 50% correct represents chance. We are interested in significant deviations from this, both above and below. The percentage correct scores were also converted to equivalent d-prime scores (right ordinate).

For statistical analysis, a percentage correct score for each image pair was derived from the three participants who saw that pair, giving *n* = 36 triplet scores per condition. To compare between all conditions we performed a Friedman’s ANOVA test, which was highly significant (*F*_*r*_(5) = 46.21, *p*<0.001), indicating that our image treatments were influencing perception of scale. Five planned post-hoc Wilcoxon signed rank tests were also performed to compare the gradient blur condition (the condition of primary interest) with each of the five other conditions. A further six Wilcoxon signed rank tests were performed to compare each of the conditions against chance. All tests were two-tailed, and we Bonferroni corrected the p-values for each set of Wilcoxon tests (i.e. we multiplied the p-values by the appropriate correction factor, so the reported values can be compared to a threshold of *α* = 0.05). The outcomes are reported below as appropriate. (The results that did not reach significance did not do so even without Bonferroni correction).

When there was no blur (red bar) the full-scale scenes were correctly identified significantly more often than chance, indicating that our participants could reliably identify the scale of the scene in the photograph when they were untreated (Z = 2.42, *p* = 0.046). That performance was markedly less than 100% is a testament to the quality of the scale model images that we were able to obtain. (See [17] for some discussion on the perception of scale models). However, when the full-scale scenes were blurred with a vertical blur gradient (green bar), performance dropped well below that for the untreated images (Z = -4.71, *p*<0.001) and well below chance (Z = -4.36, *p*<0.001) meaning that our participants systematically concluded that the small-scale model photographs looked more like full-scale scenes than the photographs of the real full-scale scenes. When the blur gradient was replaced with the square wave treatment (purple bar), the results were not significantly different from each other (Z = -1.80, *p* = 0.368), though they remained significantly below chance (Z = -2.89, *p* = 0.01). When the blur gradient was rotated through 90 deg (blue bar) the results were not significantly below chance (Z = 1.61, *p* = 0.320) and the effect was significantly less than for the original gradient blur condition (Z = -2.76, *p* = 0.029). Neither of the two control conditions were significantly different from chance (yellow and pink bars, both *p*>0.05), but performance was significantly better than in the gradient blur condition for each of them (inverse gradient blur: Z = 3.66, *p* = 0.001; full blur: Z = 3.89, *p*<0.001).

Short informal debriefing of participants revealed that most found the task to be much more difficult than anticipated. (In fact, not one of our 108 participants got all six of their trials correct). Many commented that one of the images was always blurred, but most were unclear about how or if this had a systematic effect on their behaviour.

## Discussion

Our results are consistent with a claim that the application of appropriately aligned gradients of blur causes negative d-prime for detecting the reality of scale. Presumably this happens because when the blur is placed at the top and bottom of the image this corresponds (broadly speaking) with the near and distant parts of the scene. These are the parts of the scene that would be subject to the greatest levels of optical blur in any imaging system [18]. But for the blur to be large, the imaging device would have to be rather close to the subject of the scene [31, 32], and for the breadth of field to be as large as it is, the implication is that the scene must be a small-scale model.

The generality of our conclusions might have been strengthened had it been possible to run our experiment using images in which the dominant planar surface was orthogonal to a largely hidden ground plane: the slab side of a diesel locomotive, for instance. Our informal observations confirmed that in this situation, the miniaturization effects worked well when the orientation of the blur gradient was flipped through ninety degrees (i.e., consistent with orientation of the dominant slant). However, we could find few real scene images that met this surface orientation criterion, and no appropriate photographs of models, meaning a formal investigation of this factor was not practical using our methods.

Our general conclusion above is that our image manipulation produced a tilt-shift miniaturization effect, but we should also consider other response strategies that our participants might have adopted and consider these against our results. One possibility was that participants might have systematically allocated the blurred images to one or the other of the response categories (full-scale or small-scale). However, if this had been the case, then the results for the orthogonal blur, inverse blur, and full blur conditions (right three columns in Fig 4) should have been like the gradient blur condition, but they were not. Another possibility is that the inclusion of a *linear* blur gradient was the critical factor. However, this seems unlikely (though see below) since the results for the orthogonal blur and inverse blur conditions were different from the gradient blur condition. In fact, to achieve our experimental miniaturization effects (as indicated by the negative d-prime scores) what mattered was that the blur treatment was appropriately oriented *and* appropriately signed (i.e., the blur was at the top and bottom). This is consistent with the findings of Held et al. [13] but not those of Vishwanath and Blaser [17]. However, as we outlined in the Introduction, Vishwanath and Blaser [17] provided no empirical evidence that the inclusion of a *gradient* of blur was important for the results from their type of task (there was no full blur condition, only no blur and various blur gradients) in which case, the presence of blur per se might have been the critical factor in their experiment. However, there are several other methodological differences between their study and ours (and Held et al.’s). For example, both we and Held et al. used images of complex three-dimensional scenes, whereas Vishwanath and Blaser used planar textured surfaces (rock faces), with no familiar size cues, and several poses (tilts and slants) of the surface plane compared to fairly uniform ground planes in our study and in Held et al.’s. Thus, it remains unclear what the underlying factors are for this difference in results.

The results from our square wave condition indicate that the inclusion of extended linear gradients is not strictly necessary to get the miniaturization effects; the critical factor seems to be an appropriate orientation of the contour of focus. Notwithstanding the methodological differences above, this puts constraints on theoretical proposals involving blur gradients (e.g., [17]), but does not necessarily undermine the whole idea. Indeed, it might be possible to develop a heuristic approach that would be well suited to the coarse blur conditions that we have shown to be effective. Furthermore, this coarse level of analysis is perhaps not surprising in the light of reports that (i) blur discrimination performance is ‘mediocre’ [12, 22] and (ii) that only four distinct levels of blur can be encoded in a scene [33]. Thus, it seems that although blur makes an important contribution to the perceived scale of a scene, its role in human vision might be coarser than its mathematical potential. One reason for this might be that the use of blur in the intersection of constraints proposal [13, 14] requires that the visual system knows about pupil diameter (this is the parameter equivalent to interocular distance in the case of binocular stereopsis, which is of course fixed in humans). We know of no evidence to suggest that the visual system has access to this state of its anatomy.

One possible weakness in our experimental design is that there was no control for the locations of cues to real full-scale scenes across position in the original images. For example, perhaps the details of trees are a strong cue to full-scale reality, and perhaps these are more evident at the top parts of the scene. If this were the case, then sharp giveaway cues might be more numerous in the orthogonal gradient blur condition than in the gradient blur condition and this might undermine some of our conclusions. Of course, with so many possible factors in the images, it was not realistic to try and adopt formal control of them all, though as mentioned in the Methods, we did our best to avoid using images with obvious giveaways to scale models versus full-scale scenes.

A study with some superficial similarity to ours, reportedly on scene memory, was performed by Hafri et al. [26]. They applied fake tilt-shift treatment to photographs of various scenes and required participants to perform 2AFC distance discrimination. The original images across each 2AFC pair were always the same, their only difference was the application of the tilt-shift treatment to one of them. Operationally then, the task was a blur (or blur-gradient) discrimination task. Thus, while in our experiment, the ground truth pertained to the scale of the scene, in Hafri et al’s, it pertained to the blur. Of course, in our experiment, the blur treatment (including no blur) was also applied to only one of the images in the pair so our experiment could also have been operationalised as a blur discrimination task. However, as pointed out above, this is not what our participants did. It is unclear how Hafri et al.’s participants performed the task, but they were correct on about 70% of the trials. Thus, our own experiment used tilt-shift and 2AFC to investigate perception of scale gauged against truth whereas Hafri et al’s did not.

The work here concentrated on what we refer to as the class 2 cue of blur gradients, which has been proposed to be used in an intersection of constraints with class 1 pictorial cues to provide information about scale [13, 14], or more directly, as a cue to nearness [17]. However, the work and review here suggest that while blur is used in the perception of scale, it is probably done rather crudely. Of course, other class 2 cues (horizontal binocular disparity and motion parallax) not investigated here, might also contribute to an intersection of constrains analysis, or as cues to nearness from their gradients, in a similar way to Vishwanath & Blaser’s proposal for blur, improving the system’s overall estimate of scale. Furthermore, class 2 cues can also contribute to the perception of absolute depth across the entire scene, once one or more initial estimates of absolute depth are secured (e.g., [19]). Finally, as Mather and Smith [15] pointed out, horizontal disparity and blur are most effective at different distances (e.g., blur is available beyond the range of disparity mechanisms), and hence likely to complement each other. Held et al. [34] came to a similar conclusion, though Maiello et al. [32] concluded that blur is detrimental to perception of depth when other cues are available (see also [35] for an experiment and critical appraisal).

The application of blur gradients to images has received formal treatment previously [13, 14, 17, 36] but, for a while, it also become widely known through popular media, advertising and movie making, where it is sometimes referred to as fake tilt-shift miniaturization (e.g., [16]) or simply, fake miniaturization [26]. Many compelling demonstrations of this can be found on the Internet with appropriate key words and a search engine, but the effects in most of these examples are enhanced by photographers and artists who artificially increase the colour saturation (Hafri et al. [26] treated their experimental stimuli in a similar way). There are at least two reasons why this treatment is likely to be effective. First, colour saturation is an important parameter in the depth cue known as atmospheric perspective where intervening particulate matter will make more distant objects less saturated in the retinal image (e.g., [37]). Hence, if an entire scene is saturated, the implication is that all of it is close, and therefore small. The second reason is that we speculate that toy scale models are often built from brightly coloured products that might look exciting but are not very realistic. Our familiarity with these products is likely to enhance the toy-like—and hence miniature—appearance of images processed this way. Our work here has avoided these confounds by using achromatic images.
